# CLOCKΔ19 mutation modifies the manner of synchrony among oscillation neurons in the suprachiasmatic nucleus

**DOI:** 10.1038/s41598-018-19224-1

**Published:** 2018-01-16

**Authors:** Mitsugu Sujino, Takeshi Asakawa, Mamoru Nagano, Satoshi Koinuma, Koh-Hei Masumoto, Yasufumi Shigeyoshi

**Affiliations:** 1 Department of Anatomy and Neurobiology, Kindai University School of Medicine 377-2 Ohno-Higashi, Osakasayama City, Osaka, 589-8511 Japan; 2 Information Systems Center, University of Occupational and Environmental Health, 1-1, Iseigaoka, Yahatanishi-ku Kitakyushu-shi, Fukuoka, 807-8555 Japan; 3 Center for Medical Science, International University of Health and Welfare 2600-1 Kitakanemaru, Ohtawara, Tochigi, 324-8501 Japan

## Abstract

In mammals, the principal circadian oscillator exists in the hypothalamic suprachiasmatic nucleus (SCN). In the SCN, CLOCK works as an essential component of molecular circadian oscillation, and *Clock*Δ19 mutant mice show unique characteristics of circadian rhythms such as extended free running periods, amplitude attenuation, and high-magnitude phase-resetting responses. Here we investigated what modifications occur in the spatiotemporal organization of clock gene expression in the SCN of *Clock*Δ19 mutants. The cultured SCN, sampled from neonatal homozygous *Clock*Δ19 mice on an ICR strain comprising PERIOD2::LUCIFERASE, demonstrated that the *Clock* gene mutation not only extends the circadian period, but also affects the spatial phase and period distribution of circadian oscillations in the SCN. In addition, disruption of the synchronization among neurons markedly attenuated the amplitude of the circadian rhythm of individual oscillating neurons in the mutant SCN. Further, with numerical simulations based on the present studies, the findings suggested that, in the SCN of the *Clock*Δ19 mutant mice, stable oscillation was preserved by the interaction among oscillating neurons, and that the orderly phase and period distribution that makes a phase wave are dependent on the functionality of CLOCK.

## Introduction

Our internal clock mechanism, named the circadian clock, governs daily variations in many metabolic, physiologic, and behavioral functions^1^. In mammals, the center of the circadian clock resides in the suprachiasmatic nucleus (SCN) in the hypothalamus^2,3^. About 10,000 cells are present in the unilateral nucleus of the SCN and about 20% of the cells have the ability to generate a self-standing circadian rhythm^4,5^.

The intracellular molecular oscillator is composed of interacting positive- and negative transcription/translation feedback loops of the clock gene^3,6,7^. The transcriptional activators circadian locomotor output cycles kaput (CLOCK) and brain and muscle ARNT-like protein 1 (BMAL1) form a heterodimer and stimulate the transcription of other clock genes, such as *Periods* (*Per1*, *Per*2, and *Per3*) and *Cryptochromes* (*Cry1* and *Cry2*), through binding to the E-box response elements located in the promoter region. Accumulated PER and CRY proteins form a complex that represses the transcriptional activity of the CLOCK/BMAL1 heterodimer. This autoregulatory loop builds an oscillation of gene expression approximately 24 h in duration.

*Clock*Δ19 mutant mice have a point mutation that causes a deficiency in the 19^th^ exon of the *Clock* gene^8–10^. As the mutant CLOCK protein blocks normal circulation of the feedback loops of clock gene, the molecular oscillator attenuates the SCN and becomes arrhythmic in the peripheral organs^11–14^. The mutant mice exhibit abnormally long periodicity of spontaneous activity under constant environments, and slightly decreased contrast between the light-period and the dark-period in activity and feeding under a light-dark cycle. Additionally, their phase-shifting responses to light pulses in circadian activity rhythms are much larger than that of wild-type mice^14,15^.

*In vivo* and *in vitro*, the circadian clock in the SCN of *Clock*Δ19 mutant mice exhibits long periodicity, attenuation of gene expression that depends on the E-box transcriptional cis-element, and high-amplitude phase-resetting responses^14–18^. Furthermore, SCN transplantation from wild-type mice restores stable circadian activity rhythms with normal periodicity in the *Clock*Δ19 mutant mice^19^. These studies indicated that the abnormal circadian activity rhythms in *Clock*Δ19 mutant mice stem largely from the SCN.

Circadian oscillation of the SCN depends not only on intracellular, but also intercellular processes^20^. The chemical and electrical cell-to-cell connections integrate each cellular oscillation, which creates a diverse endogenous period, and the SCN outputs a robust signal with a single period^16,21,22^. Although the oscillators in individual cells synchronize to each other, there is a regional difference in the daily oscillation of circadian gene expression in the SCN^23–28^. The rhythmic expression of the clock gene is initiated in the medial/caudal region and then propagates to the lateral/rostral region. This propagation, called the phase wave, has been monitored both *in vivo* and *in vitro* such as in organotypic slice culture^29–31^. Some reports have suggested that intercellular connection and spatial phase difference is involved in the clock function of the SCN^25,32^. For example, gene ablation of *Rgs16* impaired normal spatiotemporal organization of clock genes in the SCN, and lengthened the circadian period of behavioral rhythms^25^.

In the *Clock*Δ19 mutant SCN, the spatial profile of the circadian oscillator is not yet fully understood. To elucidate how the mutant CLOCK protein changes the SCN, we crossbred the *Clock*Δ19 mutant mice to PERIOD2::LUCIFERASE (PER2::LUC) knock-in mice^33^, and obtained hybrid mice that allow real-time monitoring of *Per2* gene expression by quantitating the amount of bioluminescence. It is reported that *Clock*Δ19 mutant mice show a different phenotype depending on the strain^34–36^. We used an ICR strain in this study, because the long periodicity of mutant mice on an ICR genetic background persists for more than one month under constant darkness^37^; however, mutant mice on a C57Bl/6 genetic background lose circadian rhythmicity within two weeks. As expected, the cultured mutant SCN from new-born mice showed a stable circadian bioluminescence rhythm. In this report, we show characteristics of the circadian rhythm of oscillators in the SCN of the *Clock* mutant mice, especially spatiotemporal organization.

## Results

### Locomotor activity of homozygous *Clock*Δ19 mutant mice containing PER2::LUC on an ICR strain

Spontaneous activity was measured in *Clock* mutant mice on an ICR genetic background entrained to 12 h-light/12 h-dark cycles, and showed a long free-running period under constant darkness (wild-type 23.9 ± 0.0 h, heterozygous 24.7 ± 0.1 h, homozygous 26.2 ± 0.2 h, Supplementary Fig. S1A and B). All the measured homozygous mice sustained circadian activity rhythms over four weeks under constant darkness. The temporal distribution of the entrained spontaneous activity rhythm is shown in Supplementary Fig. S1C. The mutant mice showed an increase in activity in the daytime (ZT 6–9) and a delay in the onset of the active period (ZT 10-17) compared with wild-type mice.

### The circadian bioluminescent rhythm generated in SCN explants from homozygous *Clock*Δ19 mice

**Figure 1:**
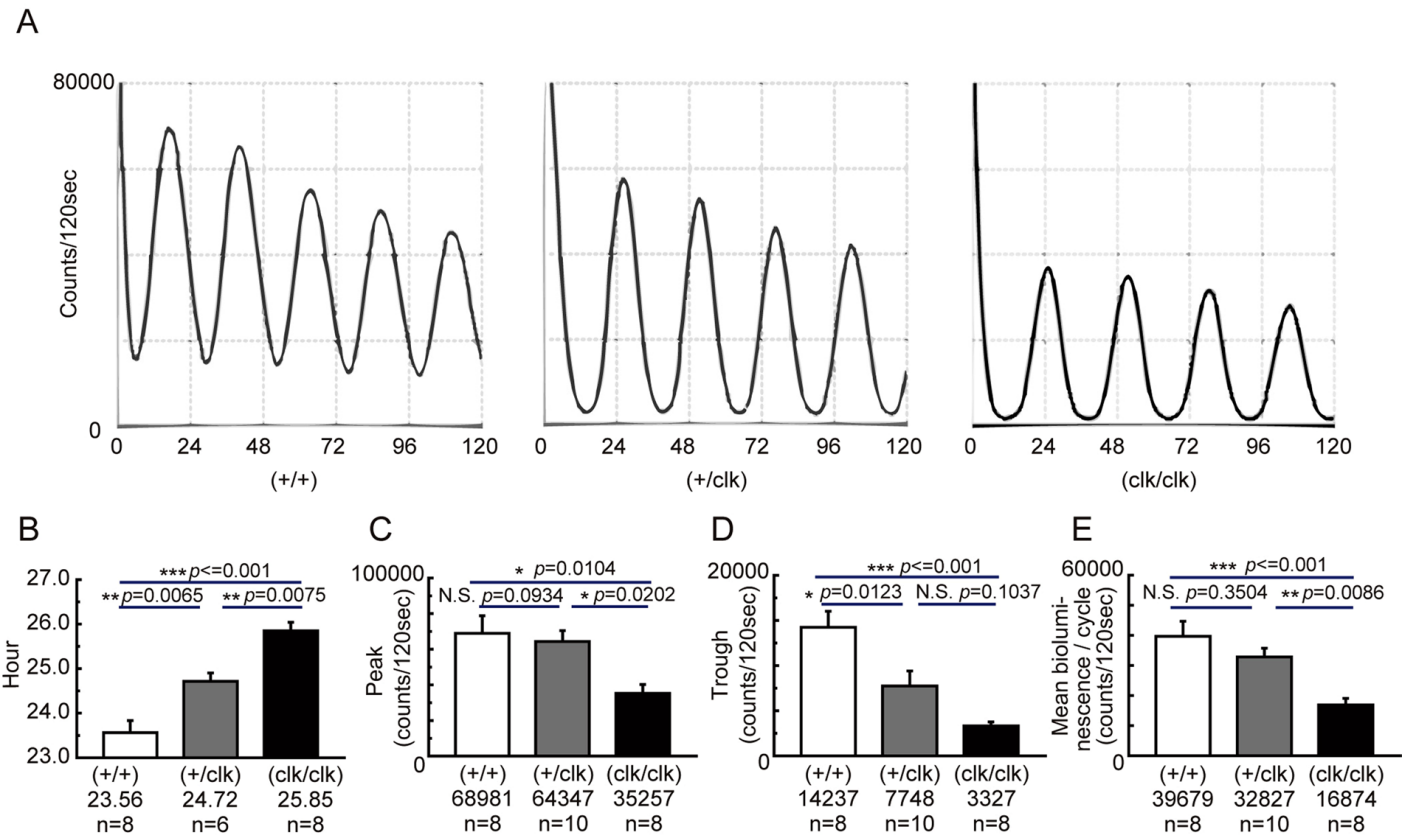
Recording of circadian bioluminescence rhythms in primary SCN cultures prepared from *Clock*Δ19 mutant new-borns containing the PER2::LUC genetic modification. (**A**) Representative traces of PMT bioluminescence recordings of wild-type (+/+), heterozygous (+/clk), and homozygous (clk/clk) *Clock* mutant SCN. The vertical axis shows the bioluminescence intensity and the horizontal axis shows the number of hours of the PMT recording. (**B**–**E**) Characteristics of PER2::LUC bioluminescence rhythms in *Clock* mutant SCN cultures. (**B**) The period length of the bioluminescence rhythms, which are defined as the mean time of the peak-to-peak between the second and fifth waves. (**C**,**D**) The mean of the peak and trough values of the bioluminescence rhythms, respectively. (**E**) The mean bioluminescence intensity per cycle. Results were estimated using the second, third, and fourth waves. Means ± SEM, Tukey-Kramer test, *p < 0.05, **p < 0.01, ***p < 0.001.

We prepared organotypic slice cultures of the SCN and measured PER2::LUC signals of cultured SCN using a PMT. The cultured *Clock* mutant SCN sampled from new-born mice showed a distinct and stable circadian bioluminescence rhythm (Fig. 1A). The homozygous and heterozygous mutant SCN exhibited significantly longer free-running periods (wild-type 23.56 ± 0.27 h, heterozygous 24.72 ± 0.19 h, homozygous 25.85 ± 0.19 h, Fig. 1B), similar to the behavioral rhythms observed *in vivo* (Supplementary Fig. S1B). Compared with those of wild-type SCN, the peak intensity of bioluminescence rhythms in homozygous mutant SCN was decreased to 51.1%, while the trough intensity of rhythms was reduced to 23.4% (Fig. 1C and D). The amplitude, which was defined as the difference between the peak and trough values, was reduced to 58.3%. However, the average bioluminescence amount per unit time in one cycle was reduced to 42.5% of that of wild-type mice (Fig. 1E). These rhythms were sustained over four weeks without changing the medium (4 of 4 slices, data not shown).

### The *Clock*Δ19 mutation impaired the spatial phase wave of *Period2* expression in the cultured SCN

**Figure 2:**
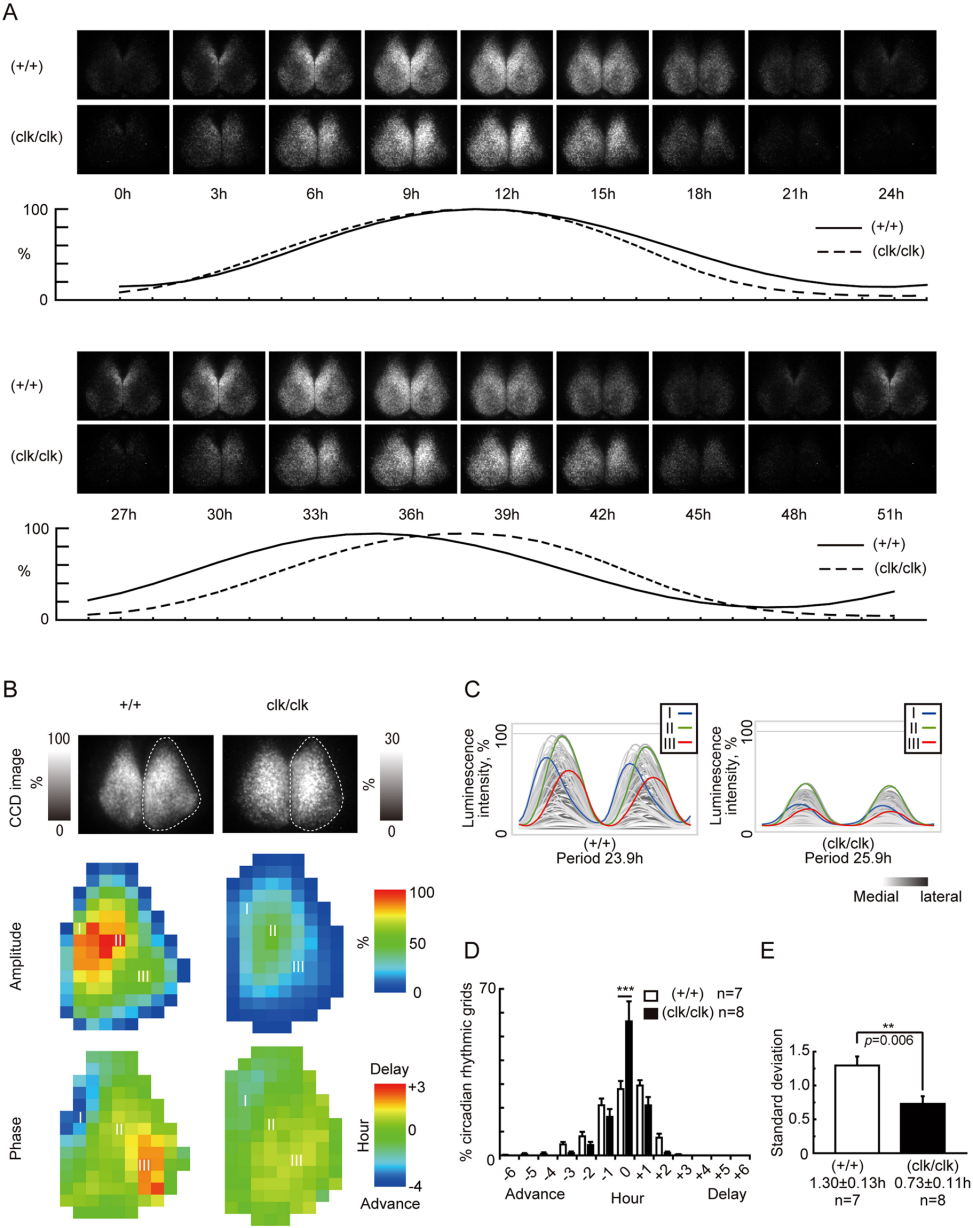
The spatiotemporal pattern of the PER2::LUC SCN for representative slices from wild-types and homozygous *Clock*Δ19 mutants. (**A**) CCD images are arranged in chronological order at 3-h intervals, and the bioluminescence intensity is shown below the pictures. The peak value of bioluminescence intensity was set to 100% in each series. (**B**) A bioluminescence image, amplitude map, and phase map of the SCN slice are shown. Characteristics of the circadian bioluminescence rhythm in each grid were obtained by a cosine-curve fitting method. The unilateral SCN (right) was analyzed, and estimated borders are shown as broken lines. In the amplitude map, the pseudo-colored images are normalized to the highest amplitude grid in the wild-type slice. The phase of each grid was normalized relative to the acrophase of the whole analyzed area, and is represented as an actual time (hour). (**C**) The signal intensity of the individual grids in (**B**) over time (2 cycles). Colored lines marked with ‘I’, ‘II’, and ‘III’ are typical grids localized in the medial lip of the dorsal region, high amplitude area of the dorsal region, and the ventrolateral region in (**B**), respectively. Data is normalized to the highest signal intensity in the wild-type slice. The brightness of each line indicates the position on the medial-lateral axis. (**D**) The distribution of the phase difference in the wild-type and *Clock* mutant SCN slices. There was a significant interaction in a two-way ANOVA (wild-type vs. homozygous mutant, p < 0.0001). Means ± SEM, Tukey-Kramer test, ***p < 0.001. The vertical axis is the percentage of the total number of the circadian rhythmic grid. (**E**) Comparison of the standard deviation of the phase difference in the SCN slices. Means ± SEM, t-test, **p < 0.01.

To investigate the spatial characteristics of circadian rhythmicity in the mutant SCN explants, we analyzed time-lapse bioluminescence images of the SCN captured by a cryogenic CCD. The explants from wild-type mice showed a dynamic circadian oscillation and phase wave, which started in the medial SCN and propagated toward the lateral region (Fig. 2A). In particular, the luminescence wave rose from the packed area of the medial lip of the dorsal SCN. On the other hand, in the *Clock* mutant SCN, the increase in luminescence started in the wider region of the medial SCN. In addition, even though the mutant SCN had a long period, the high light-emitting time was short. Therefore, the spatial phase difference was small and the phase wave became ambiguous in the *Clock* mutant SCN (Fig. 2A).

Next, we measured a more refined regional phase and amplitude distributions in the SCN explants. The series of bioluminescence images were divided into a rectangular lattice, and the phase and amplitude of oscillatory luminescence were calculated in each grid containing the SCN using a cosine curve fitting method. Figure 2B shows a representative spatial pattern of the amplitude and phase distribution in the wild-type and *Clock* mutant SCN, and the bioluminescence rhythms of each grid are shown in Fig. 2C. The amplitude of the bioluminescence rhythms in the mutant SCN was weaker than that of the wild-type across the whole SCN (Fig. 2B, middle row and Fig. 2C); however, both mutant and wild-type SCN had a similar distribution pattern. The high amplitude area is localized in the dorsal side of the medial region and is marked ‘II’, and the middle amplitude area is localized in the ventral side of the lateral region and is marked ‘III’ (Fig. 2B, middle row). The represented spatial phase map in bottom row of Fig. 2B indicates that the wild-type SCN had a broad phase wave, which flows from the medial lip of the dorsal region to the lateral side of the ventral region. From the average phase of the whole SCN rhythm, the oscillation in the leading area marked ‘I’ was early by 3.4 h, and the oscillation in the ragging area marked ‘III’ was delayed for 2.2 h. The wild-type SCN explants consisted of many regions that had various phases (Fig. 2D). The grids with the same phase (± 0.5 h) as the average phase of the whole SCN comprised only 27.9 ± 3.4% of the circadian rhythmic grids, and the most common (29.4 ± 2.2%) grid was 0.5–1.5 h behind the average phase of the while SCN. In contrast, the phase wave in the mutant SCN was narrow. The represented mutant SCN showed a spatial phase difference, but the leading grid (I) was early by only 1.5 h, and the ragging grid (III) was delayed for 0.9 h from the average phase of the whole SCN (Fig. 2B, bottom row and Fig. 2C). The distribution’s spread was smaller than that of the wild-type (Fig. 2D). More than half (56.4 ± 8.4%) of the rhythmic grids had the same phase (± 0.5 h) as the average phase of the whole SCN. A significant difference (p = 0.006) in the standard deviation of the phase variance was observed between the wild-type (1.30 ± 0.13 h) and mutant (0.73 ± 0.11 h) SCN explants (Fig. 2E).

### TTX treatment damped and desynchronized cellular circadian *Period2* expression rhythms in the cultured SCN from *Clock*Δ19 mutants

**Figure 3:**
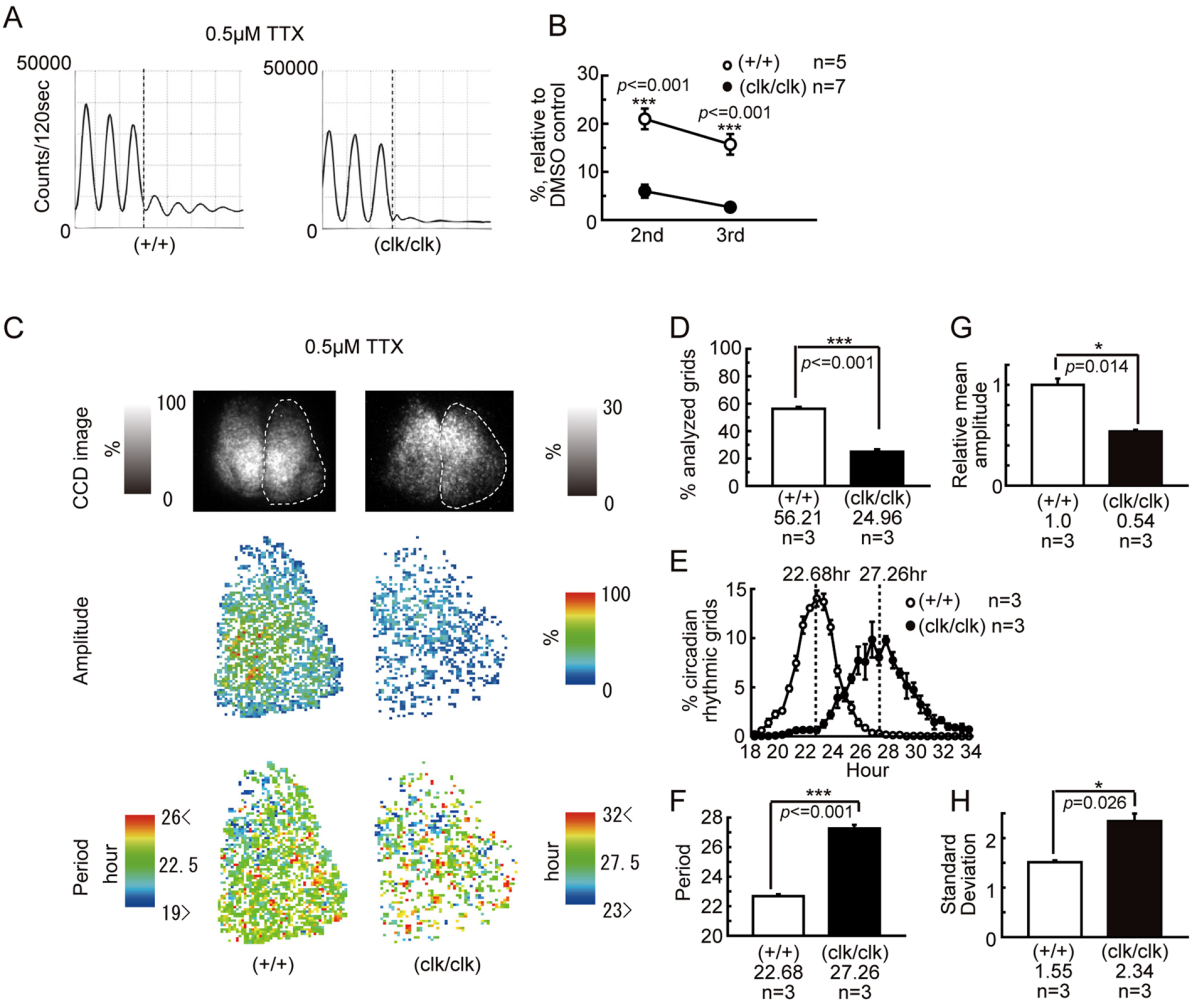
Damped and desynchronized cellular circadian PER2::LUC expression rhythms in the *Clock*Δ19 mutant SCN slice after tetrodotoxin (TTX) administration. (**A**) Effect of TTX (0.5 μM) on PER2::LUC expression in the *Clock*Δ19 SCN slice. Typical traces of PMT bioluminescence recordings of the wild-type (+/+) and homozygous (clk/clk) *Clock* mutant SCN are shown. Vertical broken lines indicate the time point of TTX administration. Damping rates of the wave amplitude after TTX administration are shown in (**B**). The vertical axis is relative to the amplitude of the vehicle control. The first wave after administration was ignored, because it showed a much higher variability than the following waves. (**C**) The spatiotemporal pattern of the cellular PER2::LUC rhythms in *Clock*Δ19 SCN slices after TTX administration. A typical bioluminescence image, amplitude map, and period map of the SCN slice are shown. The unilateral SCN (right) was analyzed, and estimated borders are shown as broken lines. The grayscale bars indicate the relative bioluminescence intensity, and a value of 100 is defined as the brightest area in the CCD image of wild-types. In the amplitude map, the pseudo-colored images are normalized to the highest amplitude grid in the wild-type slice. The period range of the pseudo-color was based on the period distribution (see (**E**)). (**D**–**H**) Statistical analysis of the amplitude and period maps in (**C**). (**D**) The percentage of the circadian rhythmic grid in all analyzed grids. In the present analysis, we defined the period range of the circadian rhythm to be between 18 h to 35 h. (**E**) The period distribution of the circadian rhythmic grid. A vertical broken line indicates the mean of the period. (**F**) Comparison of the mean period of the circadian rhythmic grid. (**G**) Comparison of the mean amplitude of the circadian rhythmic grid. (**H**) Comparison of the standard deviation of the period difference. Means ± SEM, Tukey-Kramer test in (**B**), t-test in (**D**,**F**,**G** and **H**), *p < 0.05, **p < 0.01, ***p < 0.001.

Some reports have indicated that the intercellular connection requires sustained robust circadian rhythmicity of the whole SCN^20,38^. To test the role of intercellular coupling in the *Clock* mutant SCN, we treated the SCN explants with tetrodotoxin (TTX), which desynchronizes neurons by blocking action potentials^39^. The wild-type SCN explants showed a reduction in PER2::LUC luminescence and damping of oscillations under TTX administration, but these rhythms persisted over four cycles (Fig. 3A). In contrast, the rhythmicity of the mutant SCN rapidly faded after TTX treatment (Fig. 3A). Figure 3B shows the effect on the amplitude of the luminescence rhythms after TTX treatment. The amplitude of the mutant SCN rhythm was measured for three cycles immediately after administration, but we excluded the first wave of the comparison because it showed much higher variability than the following waves. The amplitude of the second and third cycles in the wild-type SCN were decreased to 21.0 ± 2.1% and 15.7 ± 2.2% of that in the vehicle controls, respectively (Fig. 3B). In the mutant SCN, the decrease in the amplitude was more severe (second cycle, 6.0 ± 1.4%, third cycle, 2.6 ± 0.8%), and was significantly larger than that of the wild-type (second cycle, p < 0.001, third cycle, p < 0.001) (Fig. 3B). To examine the properties of each cellular rhythm under a desynchronizing condition, time-lapse SCN images were analyzed using a micro-size grid, which allowed for the calculation of rhythmicity at the single cell-level. In this study, we judged the grid to have a significant circadian rhythm (18 ≤ τ ≤ 35 h), if the multiple correlation coefficient of the fitted sine curve was 0.6 or more. Spatial period maps indicated that individual cells sustained the circadian rhythmicity in both the wild-type and *Clock* mutant SCN (Fig. 3C). However, the circadian rhythmic grids in the mutant SCN were significantly smaller than that in the wild-type SCN (p = 0.0018, wild-type, 56.2 ± 1.4%, mutant, 25.0 ± 1.8% of analyzed grids, Fig. 3D), and the amplitude was also significantly decreased (p = 0.002, Fig. 3G). The genetic background was reflected in the period of the circadian rhythmic grids (wild-type, 22.7 ± 0.1 h, mutant, 27.3 ± 0.2 h, Fig. 3F). Furthermore, uncoupled mutant SCN cells showed highly variable periods of rhythmicity (Fig. 3E and H). The wild-type SCN showed a spatial period difference, in which the cells with a shorter period were packed in the medial lip of the dorsal SCN (Fig. 3C). This aggregation of shorter period cells coincided with the short period region (SPR), which was found in the cultured rat SCN under forskolin treatment in our previous study^23^. In contrast, this SPR-like cluster was absent in the mutant SCN (Fig. 3C).

### The effect of inhibition of the cyclic AMP pathway on the cellular circadian *Period2* expression rhythms in the *Clock*Δ19 mutant SCN

**Figure 4:**
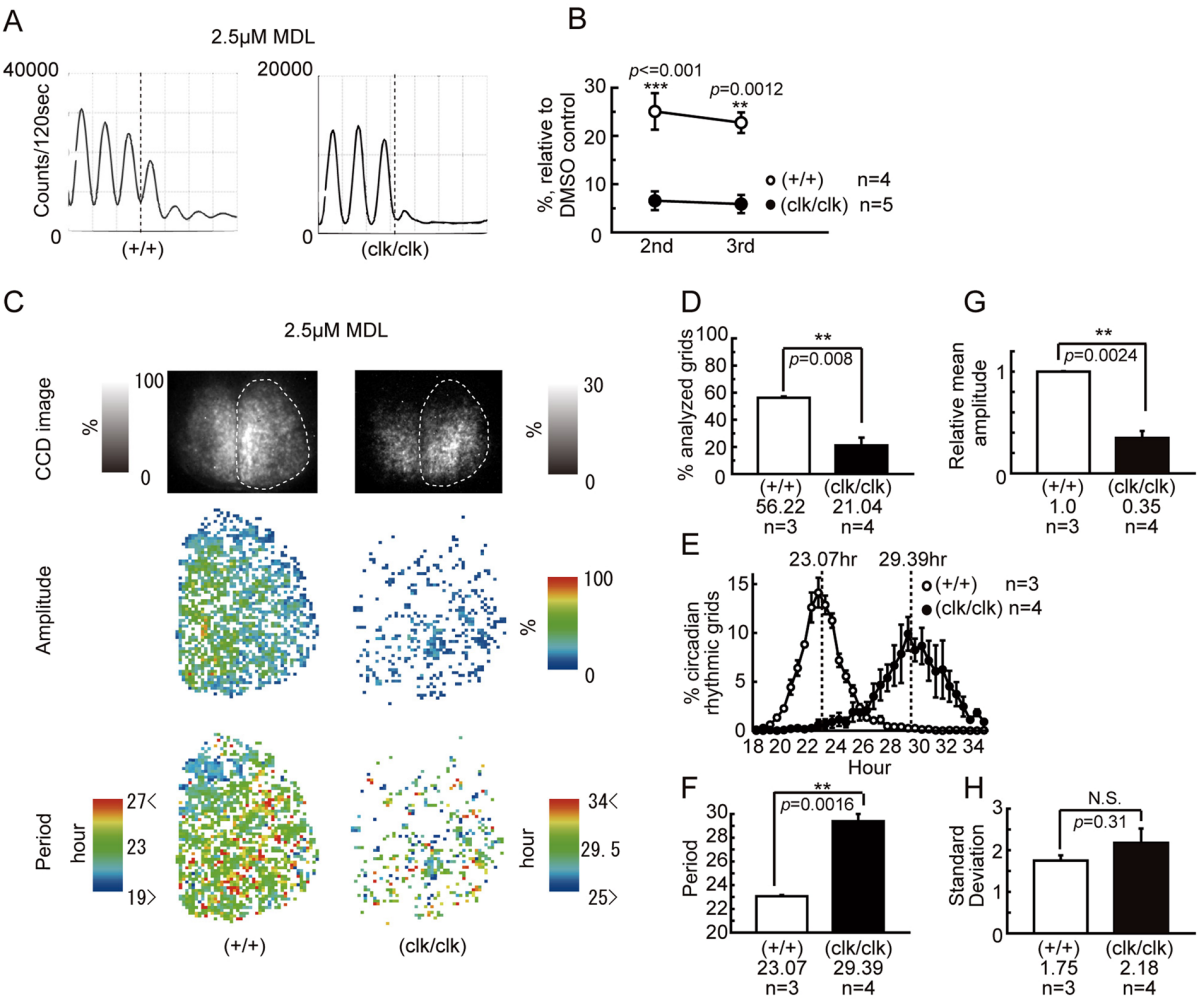
Damped and desynchronized cellular circadian PER2::LUC expression rhythms in the *Clock*Δ19 mutant SCN slice after MDL-12330A (MDL) administration. (**A**) The effect of MDL (2.5 μM) on PER2::LUC expression in the *Clock*Δ19 SCN slice. Typical traces of PMT bioluminescence recordings of the wild-type (+/+) and homozygous (clk/clk) mutant SCN are shown. Damping rates of the wave amplitude after MDL administration are shown in (**B**). (**C**) The spatiotemporal pattern of cellular PER2::LUC rhythms in *Clock*Δ19 SCN slices after MDL administration. A typical bioluminescence image, amplitude map, and period map of the SCN slice are shown. (**D**) The percentage of the circadian rhythmic grid in all analyzed grids. (**E**) The period distribution of the circadian rhythmic grid. (**F**) Comparison of the mean period of the circadian rhythmic grid. (**G**) Comparison of the mean amplitude of the circadian rhythmic grid. (**H**) Comparison of the standard deviation of the period difference. Means ± SEM, Tukey-Kramer test in (**B**), t-test in (**D**,**F**,**G** and **H**), *p < 0.05, **p < 0.01, ***p < 0.001. Explanation of these graphs is the same as described in Fig. 3.

The cyclic AMP mediated pathway constitutes a core element of intercellular connection in the SCN, and is an important activator of *Per1* and *Per2* transcription^40–42^. We investigated the role of the cAMP signaling pathway in generation of PER2::LUC rhythms in the SCN explants of the *Clock* mutant, using an inhibitor of adenylyl cyclase, MDL^43^. Similar to previous reports, the wild-type SCN showed rhythmicity over four cycles after MDL treatment, and the amplitude of the second and third cycle were 25.1 ± 3.8% and 22.7 ± 2.1% of that in the vehicle control, respectively (Fig. 4A and B). The mutant SCN showed circadian rhythmicity up to the third cycle under MDL treatment (second cycle, 6.6 ± 1.9%, third cycle, 5.9 ± 1.9%), but the decreasing rate was significantly larger than that of the wild type (second cycle, p < 0.001, third cycle, p = 0.0012). A spatial period map in the wild-type explants indicated that individual cells maintained circadian luminescence rhythms, and that the rhythmic grids were 56.2 ± 1.0% of the analyzed grids (Fig. 4C and D). As with MDL treatment, the mutant SCN had a rhythmic grid of only 21.0 ± 5.8%, which was significantly low compared with that of the wild-type (p = 0.0038, Fig. 4C and D). The amplitude of the mutant SCN cells also significantly decreased (p < 0.001, Fig. 4G). The average periods of the circadian rhythmic grids in the wild-type and mutant SCN were 23.1 ± 0.1 h and 29.4 ± 0.6 h, respectively (Fig. 4F). The variability of the periods of the mutant SCN cells was larger than that of the wild-type, but this difference was not significant (Fig. 4E and H). A spatial period map showed that the SPR-like cluster resided in the medial lip of the dorsal SCN of the wild-type (Fig. 4C). However, in the mutant SCN, the SPR-like cluster was not detected, likely because there were few grids with a significant circadian rhythm (Fig. 4C).

### Numerical simulation featuring characteristics of the circadian rhythms of the *Clock*Δ19 mutant SCN

**Figure 5:**
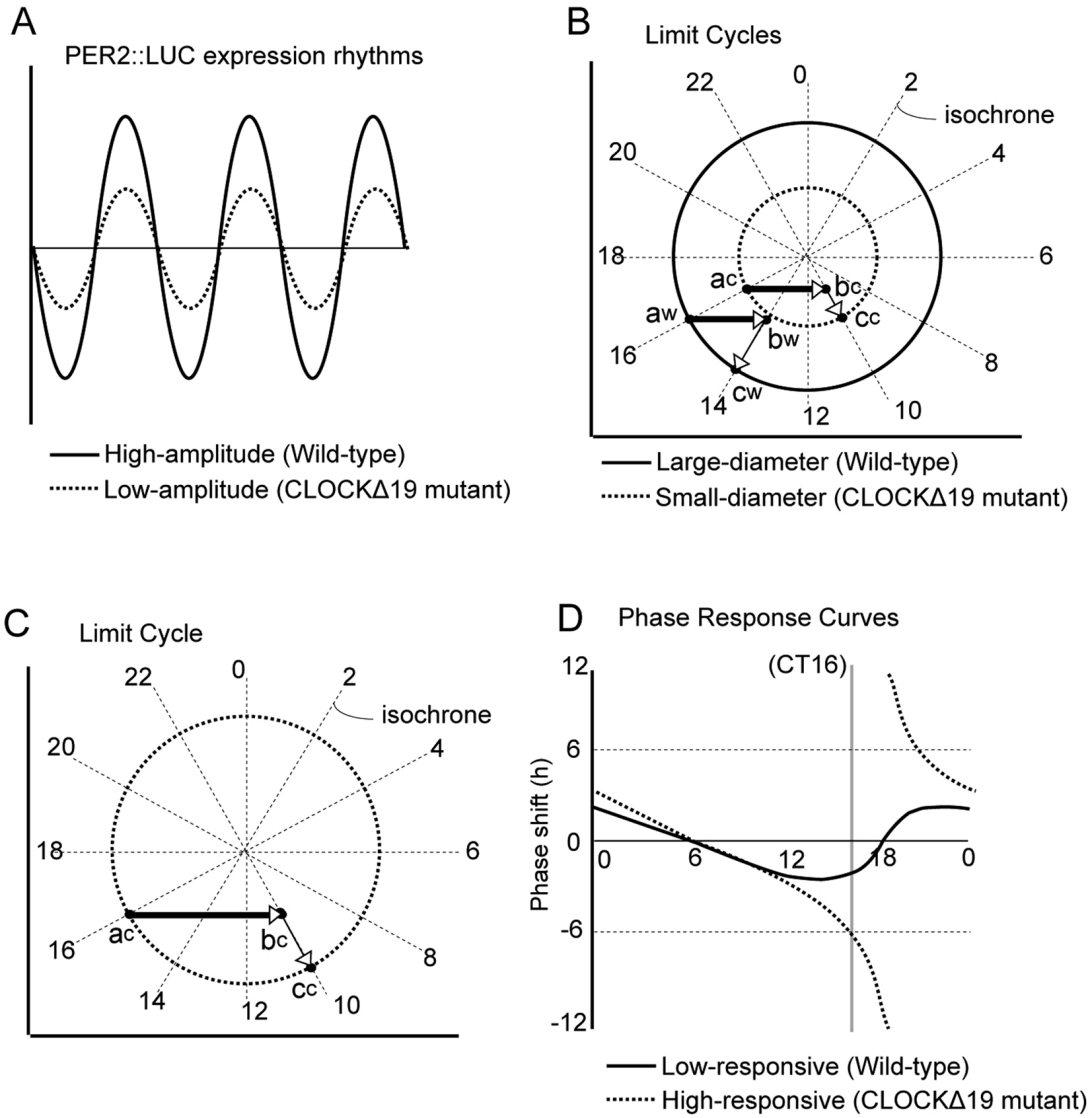
Figures shows the relationship between the length of the phase shift and the size of the limit cycle attracter. A high amplitude circadian oscillation in wild-type mice (solid-line wave in panel (**A**) is associated with a large limit cycle (solid-line circle in panel B), while a low amplitude circadian oscillation in *Clock*Δ19 mice (dashed-line wave in panel A) is associated with a small limit cycle attractor (dashed-line circle in panel B). (**B**) Shows that the same magnitude of perturbation generates a different amount of phase shift. Here, isochrones were indicated by radial lines. When perturbation, indicated by the vectors (thick arrows), moves the state variable on the limit cycle attractor to outside of the attractor, the point at which they relax back to the attractor can be determined by isochrones. In the large-diameter limit cycle, the perturbation given at point “a^w^” moves the state variable to point “b^w^” and relaxes back to point “c^w^” on the limit cycle. This causes a 2-hour phase delay (CT16 to CT14). On the other hand, the same perturbation elicits a 6-hour phase delay (CT16 to CT10) in the smaller limit cycle (from a^c^ to b^c^ to c^c^). (**C**) Even with a larger limit cycle, equivalent amount of the phase shift is attained by a stronger perturbation (a^c^ to b^c^ to c^c^). (**D**) Generally, at the same magnitude of the perturbation, the low-amplitude oscillator has a high-magnitude phase response curve (PRC of dashed-line) compared with the high-amplitude oscillator (PRC of solid-line). CT is circadian time.

Given these results, we suggested that the spatiotemporal characteristics of *Clock* mutant SCN cells consist of four features: (a) loss of localization of short period oscillators (SPOs), (b) reduction of the amplitude, (c) period extension, and (d) increase of period variability. In order to investigate what kind of influence these factors had on the spatial phase difference in the *Clock* mutant SCN, we performed a numerical simulation designed by a two-dimensional grid, in which each grid has an independent oscillator. Here, we used a phase equation to describe the circadian limit cycle oscillators^44^. Since the phase equation does not contain information about the amplitude, we represent the lower amplitude of the *Clock* mutant mice as an increase in the coupling constant. The degree of the shift is determined by the relative size of the limit cycle attractor and perturbation when the perturbation was given during the same phase (Fig. 5)^15,45,46^. Thus, the decrease in the amplitude of the limit cycle with a fixed magnitude of perturbation is relatively equivalent to the increase in the magnitude of perturbation with a fixed amplitude of the limit cycle. Variations in this condition often produce a change from type1 to type0 phase response curves, which indicates a stronger interaction between oscillators that is described as increase in the coupling constant in the present phase equation model.

Therefore, we simulated the following cases: (1) changing the concentration rate of SPOs in the SPR, (2) changing the coupling constant *g*, which defines the strength of cell-to-cell coupling. This condition corresponds with alteration in decrease in the amplitude of circadian rhythm. (Fig. 5) (3) changing the synchronized period (T = 23.1~28.1) by shifting all inherent periods of each oscillator, and (4) changing the random selection of the inherent period with a different standard deviation (*σ* = 1~3).

**Figure 6:**
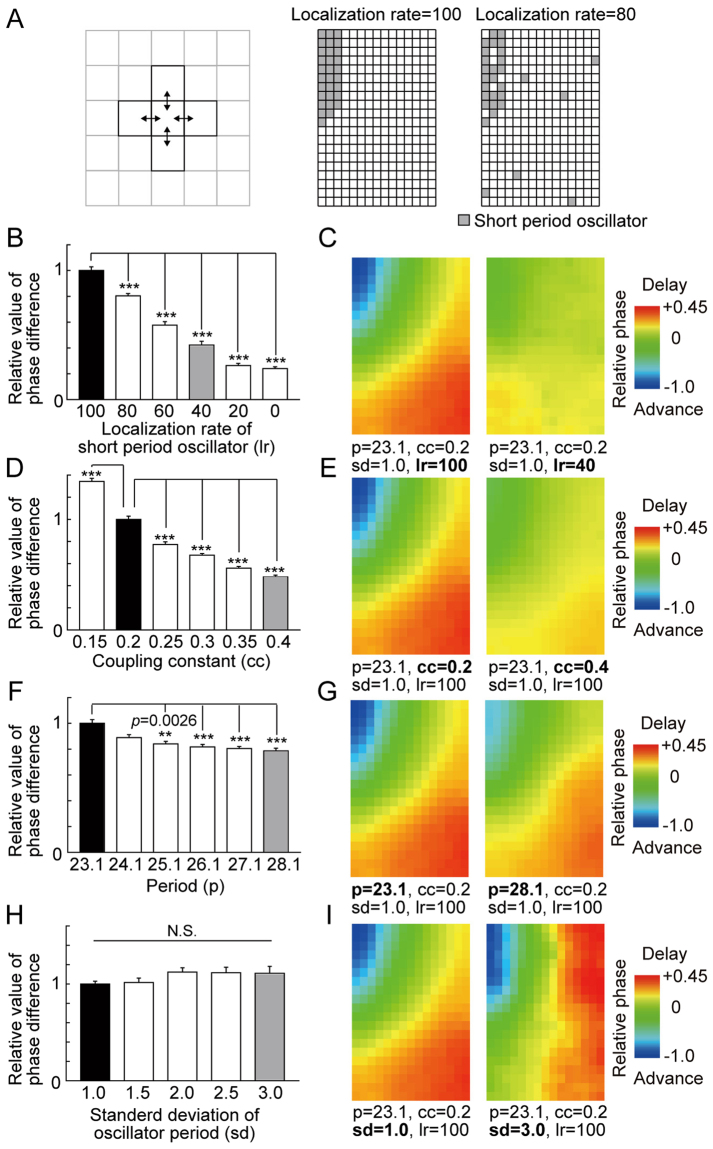
Numerical simulation of the phase pattern. (**A**) A schematic representation showing the localization of interactions between the central and surrounding grid elements. In the present model, the interaction was limited to directly neighboring oscillators. A schematic representation showing the arrangement of grid elements representing limit cycle oscillators. The short period oscillators (SPOs) were located in the short period region (SPR), which was in the medial small area. In cases where the localization rate was 80%, 20% of SPOs were arranged outside of the SPR at random. (**B**,**D**,**F** and **H**) Alteration to the phase difference by changing the localization rate of the SPR (lr), coupling constant (cc), synchronized period (p), and variance of each oscillator period (sd), respectively. Data is represented as the relative value to the standard condition (lr = 100, cc = 0.2, p = 23.1, sd = 1.0, black bar). (**C**,**E**,**G** and **I**) A representative pattern of the spatial phase difference of (**B**), (**D**), (**F**), and (**H**), respectively. The left and right heat maps correspond to the black bar (standard condition) and gray bar, respectively. Means ± SEM, Steel-Dwass test, *p < 0.05, **p < 0.01, ***p < 0.01.

Results of these simulations suggested that all the examined factors affected the formation of the spatial phase difference in the SCN. The simulation results can be summarized as follows; (1) The smaller the concentration rate of SPOs was, the smaller the width of the spatial phase difference became, until finally the obvious wave became unclear (Fig. 6B and C). (2) As the coupling constant became bigger, the width of the spatial phase difference decreased (Fig. 6D and E). (3) As the inherent period of each oscillator was made longer under the condition of the identical coupling constant, interestingly, the width of the spatial phase difference tended to become narrower (Fig. 6F and G). (4) When the inherent period of each oscillator with a larger standard deviation was selected, the spatial pattern of the phase wave was distorted (Fig. 6I); however, the width of phase difference was not significantly changed (Fig. 6H).

Recently, Abel *et al*. reported that the neural network of the SCN forms a Small-World Network (SWN)^47^. In order to investigate this effect in our model, we introduced the SWN to our phase equation model and investigated how the state of the synchronization was modified (see supplementary materials and methods). The results of simulations indicated that coupling through the SWN affected the phase difference in our model. As the coupling constant of the SWN increased, the phase difference decreased (Supplementary Fig. S2D). To confirm the representative results shown in Supplementary Fig. S2, we constructed 20 different SWNs with similar average path lengths and clustering coefficients. We found that all the networks demonstrated a similar trend to that shown in Supplementary Fig. S2D (data not shown). When the coupling force of the nearest neighbor was too weak to synchronize, the SWN facilitated the coupling and synchronized the two-dimensional oscillator array (Supplementary Fig. S2E). The SWN generally worked to decrease the phase difference associated with the phase waves, and alterations in the phase difference to the fluctuation of coupling constants of the nearest neighbor, localization of short period oscillators, and periods of oscillators were similar to conditions without the SWN (Supplementary Fig. S2F–H). On the other hand, the effect of shortening of the phase difference by the SWN was weak when the standard deviation of the inherent period was large (Supplementary Fig. S2I).

## Discussion

As previously reported^36,37^, the homozygous *Clock*Δ19 mutant mice on an ICR strain containing PER2::LUC generated a stable circadian rhythm with a free running period much longer than that of wild-type mice (Supplementary Fig. S1). When cultured, the neonatal SCN of the *Clock*Δ19 mutant mice generated a stable circadian bioluminescence rhythm, of which the period was longer than that of wild-type mice; however, the spatiotemporal wave, or the phase wave, was obscured (Figs 1 and 2). When the intercellular communication that maintains synchrony among oscillation neurons in the SCN was blocked, the oscillation of the whole SCN and individual cells showed much lower circadian bioluminescence rhythms (Figs 3 and 4). These findings suggest that the SCN is equipped with a molecular system of intercellular communication that compensates for the dysfunction of the CLOCK protein, which is an essential component for circadian transcriptional translational feedback.

The circadian bioluminescence rhythm generated in the SCN of *Clock* mutant mice showed a distinct pattern of circadian rhythmicity (Fig. 1). The whole SCN bioluminescence recorded using a PMT showed a lower intensity both at the peak and the trough of the circadian rhythm, which was consistent with previous reports showing that *Per1* and *Per2* expressions were reduced in the SCN of mutant mice *in vivo*^15,37,48^. Interestingly, the decrease in the trough value was much larger than that of the peak value (Fig. 1C and D). Spatiotemporal analysis using a CCD camera revealed that this characteristic of the *Clock* mutant SCN was caused by a reduction in the phase differences of the circadian rhythm among individual cells (Fig. 2). This reduction in the phase difference was associated with the modification of the phase wave propagation (Fig. 2B–E).

In the present study, the circadian oscillation of individual neurons in the SCN was attenuated when they lost synchrony after TTX or MDL treatment (Figs 3 and 4). This finding suggests that the cellular interaction that maintains the synchrony of oscillating neurons in the SCN also enhances and stabilizes oscillations in the SCN of the *Clock* mutant mice. This hypothesis is consistent with previous studies. Dispersed cell cultures taken from *Per1*-null or *Cry1*-null SCN did not maintain a robust circadian oscillation, but organotypic cultures of these SCN had a stable circadian rhythm^20^. The SCN explants of *Cry1*/*2* double knockout mice showed a stable circadian rhythm during the neonatal period, and this rhythm disappeared immediately in a desynchronous state caused by TTX treatment^38^. Together with the previous reports, our study suggests that there are SCN-specific mechanisms that up-regulate and maintain the oscillation, but that are not involved in the formation of the phase wave among oscillators in the SCN.

The suppression of cAMP production by MDL resulted in widespread desynchrony of oscillators in the SCN of both wild-type and *Clock* mutant mice (Fig. 4). Therefore, it is highly probable that the synchronizing mechanism that plays a major role in maintaining the circadian rhythm in the *Clock* mutant SCN is mediated by cAMP. Previous studies showed that modification of the intracellular cAMP concentration causes marked desynchronization of oscillating neurons^23,40,49^. Increase of intracellular cAMP increases *Per1* and *Per2* expression via a cAMP-responsive element situated on the promoter of the *Per1* and *Per2* genes^41,42^. *Per1* and *Per2* gene induction increase the amplitude of the circadian oscillation^50,51^, and this *Per* gene induction simultaneously shifted the phase of the circadian rhythm to fix the phase relationship among the circadian oscillators in the SCN. What triggers the increase in cAMP production? The VIP receptor signaling cascade, which activates cAMP-CREB mediated induction of *Per* transcription, is indispensable for coupling among SCN cells^22,49^. The VPAC2 receptor, which plays a major role in VIP signaling to maintain synchrony^22^, is expressed throughout the SCN, while VIP is densely expressed in the ventrolateral SCN in mice^52^. Therefore, it is likely that the dorsomedial SCN receives the VIP signal from the ventrolateral SCN, and that a once a day VIP signal is sufficient to maintain synchrony of dorsomedial SCN oscillating neurons.

The spatiotemporal pattern of the bioluminescence rhythm in the wild-type SCN took a wave form called a phase wave^29,31,53^, which flows from the medial to the lateral region. On the other hand, *Clock* mutant mice showed an ambiguous progression of the phase wave, in that the SCN did not show strong bioluminescence in the medial lip of the dorsal region, and instead started from the dorsal medial wider area. The phase wave ends in the lateral region of the SCN, but the wave terminates earlier than that of wild-type mice. What factors modified the generation of the phase wave in the SCN of *Clock* mutant mice? TTX and MDL treatment caused desynchrony among the oscillating neurons in the SCN and revealed individual profiles of the cellular circadian oscillation in the SCN. We utilized a numerical simulation based on this observation to investigate which properties cause the modification of the phase wave in the SCN of *Clock* mutant mice. Previously, according to our observation that, when the oscillators in the rat SCN were desynchronized, short period oscillators (SPOs) were densely found in a small medial lip of the dorsal region named the “short-period region (SPR)”, we proposed the hypothesis that the periodic difference between this SPR and other regions forms the phase wave^23^. In the present study, the wild-type mouse SCN showed that SPOs after TTX or MDL treatment were localized in similarly to SPRs in the rat SCN. In the *Clock* mutant SCN, compared with the wild-type SCN, the neurons showing a shorter period were dispersed widely and did not show a clear cluster of SPOs. The numerical simulation suggested that the dispersion of SPOs decreases the phase difference (Fig. 6B and C). Therefore, the absence of clustered SPOs possibly contributes to the decrease of the phase difference and the obscurity of the phase wave in the *Clock* mutant SCN. However, the possibility still remains that there is a SPR in the *Clock*Δ19 SCN. Because the bioluminescence intensity drastically decreased in the mutant SCN under a desynchronizing condition, it is possible that the SPR only became undetectable.

Other than the dispersion of the SPOs, the circadian rhythm in the SCN of the *Clock* mutant mice showed a reduction of the amplitude, an extension of the average circadian period, and an increase in the deviation of the circadian period (Figs 3 and 4). Our present numerical simulation indicated that an increase in the coupling constant decreased the spatial phase difference (Fig. 6D and E). In *Clock* mutant mice, it is hypothesized that there is a decrease in the amplitude of the circadian oscillation in oscillator neurons in the SCN, whereas the interaction between the oscillators is not attenuated. In the limit cycle model, the amount of phase shift caused by a moderate strength of perturbation is larger when the diameter of the limit cycle is smaller (Fig. 5). Therefore, it is possible that a decrease in the amplitude of the circadian rhythm shown by *Clock* mutant mice caused a smaller phase difference in the circadian rhythms of oscillators neurons in the SCN.

Furthermore, in our simulation, the phase difference was decreased along with the elongation of the circadian period of the limit cycle (Fig. 6F and G). It is also possible that the elongation of the circadian period contributed to the reduction of the phase difference. In addition, a decrease in the expression of arginine vasopressin (AVP) in the SCN contributed to a decrease in the phase difference in the SCN. The expression of AVP mRNA is decreased in the *Clock*Δ19 mutant SCN^18^, possibly due to impairment of CLOCK/BMAL1-dependent transactivation. Yamaguchi *et al*. demonstrated that mice deficient of both Vasopressin V1a and V1b receptors show decreases in the phase difference among the oscillations in the SCN and resistance to jet lag^32^. The findings support our observations of the SCN of *Clock* mutant mice. Homozygous *Clock*Δ19 mutant mice also show rapid recovery from jet lag^14,15^, which is consistent with the idea that AVP/AVP receptor interaction controls the phase difference among oscillating neurons in the SCN.

In this study, we used a phase equation model to investigate how the characteristics of the circadian rhythm in the cells in the *Clock*Δ19 mutant SCN modulate the regional phase difference. On the other hand, we have not investigated the relationship between the mutation of *Clock* gene and the spatiotemporal organization of circadian rhythms in the SCN. For this purpose, biophysical models are more appropriate to investigate the effect of *Clock*Δ19 on the spatiotemporal organization of rhythms. Kim and Forger proposed a single cell model that numerically simulated the *Clock*Δ19, and this model has been applied to the coupled oscillators^54,55^. Furthermore, a biophysical model of the population of oscillating neurons in the SCN coupled by an activator substance showed that the structure of the circadian feedback markedly affects the circadian periodicity^56^. In the future, numerical models will be established to reproduce the relationship between the *Clock* gene mutation and the spatiotemporal organization of circadian rhythms in the SCN.

Interestingly, the *After-hours* mutant mice, another model of the genetically weakened molecular clock, displayed a reduction in cell-to-cell coupling in the SCN, while their circadian period was extended, amplitude attenuated, and phase-resetting responses enhanced^57,58^. This phenotype is similar to that of *Clock* mutant mice; however, the *After-hours* mutant mice lost synchrony among neurons generating circadian oscillations in the SCN^58^. What caused the difference in the SCN of *Clock*Δ19 and *After-hours* mice in spite of the similar phenotype characterized by weak circadian rhythms? Although it is speculative, the modulation of CRY may contribute to the difference in the state of coherence. The *After-hours* mutation was mapped to the *Fbxl*3 gene, which is necessary for the degradation of CRY protein; thus, this mutation delayed the rate of CRY protein degradation^57^. CRY1 inhibits accumulation of cAMP in response to G protein–coupled receptors (GPCR) coupled to Gsa, such as the VPAC2 receptor^59^, an essential receptor to maintain synchrony among oscillating neurons in the SCN^22^. Therefore, it is possible that, in *After-hours* mutant mice, modulation of CRY1 by mutation of the *Fbxl*3 gene may disrupt the synchrony. Additional studies will be required in the future to further elucidate the mechanisms that link the circadian core feedback loop and cell-to-cell coupling.

Recently, Hong *et al*. suggested there are two types of coupling associated with the synchronization and phase wave in the SCN: phase-synchronization networks of long-range neuronal connection and diffusively propagating phase waves^60^. Abel *et al*. reported on the SWN characteristics in the manner of synchronization among neurons generating circadian rhythm in the SCN^47^. The present numerical simulation demonstrated that the SWN, which is a long-range neuronal connection, is more capable of synchronizing circadian oscillators and decreasing the phase difference among oscillators (Supplementary Fig. S2). This effect was also confirmed by 20 different SWNs with similar average path lengths and clustering coefficients. Thus, it is highly likely that the enhancement of coupling decreases the phase difference regardless of the pattern of linkage. In the *Clock* mutant mice, the SWN might contribute to the decrease in the phase difference associated with the phase wave.

## Materials and Methods

### Animals

The *Clock*Δ19 mutant mice (C57BL/6J-*Clock*^m1Jt^/J, Stock no. 002923) and Jcl:ICR mice were purchased from The Jackson Laboratory (Bar Harbor, ME) and Crea (Tokyo, Japan), respectively. The PER2::LUC knock-in mice generated by Yoo *et al*.^33^ were provided by Ueda HR. (Department of Systems Pharmacology, Graduate School of Medicine, University of Tokyo, 7-3-1 Hongo, Bunkyo-ku, Tokyo, Japan). The *Clock* heterozygous mice of the N4F1 (ICR backcrossed) generation were bred into the PER2::LUC knock-in line, and F1 offspring were backcrossed into the ICR line five times. *Clock*Δ19 and PER2::LUC double mutant mice at the N5 (ICR backcrossed) generation were intercrossed and their offspring were used in this study. All *Clock* mutant mice carried the homozygous PER2::LUC gene. All mice were housed in plastic cages (170W × 240D × 125H mm, Crea, Tokyo, Japan) under a 12-h light/12-h dark cycle (LD) under a constant temperature (23 °C). The illumination intensity was approximately 300 lux. Each cage was equipped with a water bottle and rodent laboratory chow pellets (CE-2, Crea, Tokyo, Japan). All experiments were approved by the Committee of Animal Care and Use of Kindai University School of Medicine, and all experimental procedures were conducted in accordance with the Kindai University School of Medicine guidelines for use of experimental animals.

### Behavioral Analysis

Male mice were individually housed in cages. Each cage was equipped an infra-red area motion sensor (NaPiOn, Panasonic, Osaka, Japan), which detects spontaneous movements of the animal in the cage. Locomotor activity data was recorded on an IBM-PC and then analyzed with a locomotor activity analysis program (Clock Lab, Actimetrics, Wilmette, IL). The period of circadian activity rhythm was assessed for one week from twelve days after transfer to a constant dark condition (DD) using a chi-square periodgram. The temporal distribution of entrained activity was calculated from the average of the final two days under the LD condition before transfer to the DD.

### Slice preparation

Pups (6–8 days old) were frozen to death using crushed ice, and then their brains were quickly removed and immersed into ice-cold Hank’s Buffered Saline (HBS). Tissue slices were prepared using a micro slicer (DSK-1000, DOSAKA EM, Kyoto, Japan). The brain was frontal sectioned at 300 μm, and the hypothalamic slice containing the central part of SCN was obtained. Then, the bilateral SCN was dissected from slice under a stereomicroscope (Olympus SZ61; Olympus, Tokyo, Japan) and placed on a culture membrane (Millicell-CM PICM0RG50, Merck Millipore, Darmstadt, Germany) in a 35 mm petri dish. The preparation up to here was performed in ice-cold HBS to prevent tissue damage. The SCN slices were cultured in air at 37 °C with 1.2 mL of medium that consisted of DMEM (Gibco, Carlsbad, CA), B-27 supplement (Gibco, Carlsbad, CA), 100 U/mL penicillin, and 100 μg/mL streptomycin. Approximately one week later, the explants were transferred to apparatuses for bioluminescence measurement.

### Bioluminescence monitoring

The SCN explants were transferred to the culture medium containing D-luciferin potassium salt (200 μM, Wako, Tokyo, Japan) and sealed using Parafilm to prevent evaporation. Then, the luminescence emission of the whole explants was examined using a photomultiplier tube (Kronos, ATTO, Tokyo, Japan) for 120 sec at 20 min intervals. Data analyses were performed using the Kronos Data Analysis Software and Microsoft Excel. After background subtraction, the raw data was smoothed by the moving average method. The first cycles of the recorded bioluminescence rhythm were excluded from the analysis because they showed transient increases of the amplitude, possibly induced by medium change, but not by the endogenous circadian oscillation mechanism. Peak and trough values were estimated using the mean value of the second, third, and fourth wave. The period was defined as the mean time of the peak-to-peak between the second and the fifth wave. For imaging analysis, SCN explants were installed on the stage of an inverted microscope (IX71, Olympus or ECLIPSE Ti, Nikon, Japan) equipped with a stage heater (37 °C) (TP-110R05 or TP-108R05, Tokai-Hit, Shizuoka, Japan). The bioluminescence was collected via an objective lens (x10), and images were captured every hour with a 59-min exposure by a cryogenic CCD camera (CoolSnap HQ2, PHOTOMETRICS, AZ or Clara, ANDOR, Belfast, UK) with a C-mount adaptor. CCD imaging was performed using 2 × 2 binning of the 1,392 × 1,040 pixel array and an Extended NIR mode. The camera settings were determined for the purpose of enabling measurement of cellular bioluminescence rhythms in the *Clock*Δ19 mutant SCN, based on previous reports^23,29^. Raw images were subtracted from a dark frame and filtered by a 3-dimensional median method for reduction of noise (ImageJ, Biomedical Imaging Group, NIH). Two hand-drawn adjacent regions of interest (ROIs) were specified in the left and right SCN, and then ROI containing the unilateral SCN was divided into a 2-dimensional grid array to analyze spatiotemporal characteristics. The time series of the bioluminescence intensity of the ROI was processed to remove long-term trends, and then the period, phase, and amplitude in specified time window were estimated by the cosine curve fitting method using a Visual Basic macro in Microsoft Excel^23,61,62^. To determine the spatial phase distribution in Fig. 2, the final size of each grid used was 61.5 μm × 61.5 μm, and the time window was defined as the second and third waves (between the second and forth troughs). Cosine curves were fitting for each grid using the period of the unilateral SCN that included the target grid. The spatial period distribution in Figs 3 and 4 was obtained using finer grids (10.25 μm × 10.25 μm), and the time window was 90 h, beginning 12 h after setup on the microscope. The significance of rhythmicity was determined based on the probability (*P* < 0.01) and multiple correlation coefficient (R^2^ > 0.6) of the fitting.

### Drug treatment

0.5 mM Tetrodotoxin (TTX, Wako, Osaka, Japan) and 5 mM MDL-12,330 A (MDL, Sigma, MO) stock solution was made with saline and dimethyl sulfoxide (DMSO), respectively. The final concentration was 0.5 μM for TTX and 2.5 μM for MDL. The bioluminescence rhythm of the SCN explant was recorded for one week to establish a baseline recording, and then the drug was administrated into the culture medium. The dumping rate was defined as the percentage of the wave amplitude (difference between the trough and peak values) of the vehicle control after drug administration. For image analysis, after one week, treated explants were transferred to a microscope with a cryogenic CCD camera to record the bioluminescence image.

### Mathematical simulation

Our mathematical model is basically the same as a previous suggested model^23,31,44^. The circadian rhythm has been associated with limit cycle oscillators^63–65^, and each limit cycle oscillator can be expressed by a phase equation^44^. To analyze the behavior of a large set of coupled oscillators, a phase equation model has been often used to describe a group of limit cycle oscillators. The cultured SCN slice is, in our simulation, designed by a two-dimensional grid, in which each lattice represents one oscillator. Since we are interested in the phase in such oscillators, each limit cycle oscillator is described by a phase oscillator as follows;

1 $$\frac{d{\phi }_{i}}{dt}={\omega }_{i}+g\sum _{k}\,\sin ({\phi }_{k}-{\phi }_{i})$$

2 $${\omega }_{i}=\frac{2\pi }{{T}_{i}}$$

where *φ*_*i*_ and *T*_*i*_ represent the phase and the inherent period of the *i*-th oscillator, respectively, and *g* is a coupling constant with other oscillators. We assume that one oscillator couples only with its nearest neighbors, and that there is no ‘global’ coupling; that is, there is no long-range interaction. Three hundred oscillators on a rectangular lattice of 15 columns × 20 rows were set as a two-dimensional sliced SCN model. In order to integrate the spatial period difference into our mathematical model, 30 grids with shorter period oscillators were assigned to one side and corner (Fig. 6A), named the short period region (SPR). The phase difference is determined by the difference between the fastest and the latest wave after synchronization. Under the condition of each case, simulation is performed for 20 trials, and the phase difference (radian) was converted into actual time (hour) on the basis of the synchronized period. In each trial, the inherent period of each oscillator was randomly selected, where we assumed that the period was normally distributed. The difference for the expected value between the periods of the short-period and long-period oscillators was fixed at three hours.

## Electronic supplementary material

Supplementary Information
