# Supplementary material for: CLOCKΔ19 mutation modifies the manner of synchrony among oscillation neurons in the suprachiasmatic nucleus
Source: Sci Rep. 2018 Jan 16;8:854. doi: 10.1038/s41598-018-19224-1 (PMC5770461; doi:10.1038/s41598-018-19224-1)
Supplement: Supplementary file 1 — Supplementary Information [file 41598_2018_19224_MOESM1_ESM.pdf]

Supplementary Information

Full title: CLOCKΔ19 mutation modifies the manner of synchrony among oscillation neurons in the suprachiasmatic nucleus.

Authors and affiliations:

Mitsugu Sujino[1*], Takeshi Asakawa[2], Mamoru Nagano[1], Satoshi Koinuma[1], Koh-Hei Masumoto[3], and Yasufumi Shigeyoshi[1*]

[1] Department of Anatomy and Neurobiology, Kindai University School of Medicine 377-2 Ohno-Higashi, Osakasayama City, Osaka 589-8511, Japan

[2] Information Systems Center, University of Occupational and Environmental Health, 1-1, Iseigaoka, Yahatanishi-ku Kitakyushu-shi, Fukuoka, 807-0804, Japan

[3] Center for Medical Science, International University of Health and Welfare 2600-1 Kitakanemaru, Ohtawara, Tochigi, 324-8501, Japan

Corresponding authors:

Mitsugu Sujino and Yasufumi Shigeyoshi

Department of Anatomy and Neurobiology, Kindai University School of Medicine

377-2 Ohno-Higashi, Osakasayama City, Osaka 589-8511, Japan

Tel: 8172-366-0221 (ext.3243)

Fax: 8172-368-1031

E-mail to MS: sujino@med.kindai.ac.jp

E-mail to YS: shigey@med.kindai.ac.jp

Supplementary Materials and Methods

Mathematical simulation with the Small-World Network.

A sliced SCN is mathematically modeled as a two-dimensional lattice, in which one grid represents a limit cycle oscillator. The Small-World Network (SWN) connection is added to the model used in Figure 6. According to the report by Abel et al., parameters used to characterize the SWN, i.e. the average path length (*L)* and mean clustering coefficient (*C)*, defined below[1], were experimentally measured, and *L* and C were reported to be 3~4 and 0.3~0.4, respectively. *L* is calculated by the average of the shortest paths between all pairs of nodes, d_{ij} as follows;

$$L = \frac{1}{N(N-1)} \sum_{\substack{i,j=1 \\ i \neq j}}^{N} d_{ij} \tag{1}$$

C is calculated as follows;

$$C = \frac{1}{N} \sum_{i=1}^{N} C_i \tag{2}$$

with local Clustering coefficient C_i for the *i*-th node

$$C_i = \frac{2L_i}{k_i(k_i - 1)} \tag{3}$$

where L_i represents the number of links between the k_i neighbors of node *i* (ref.). An example is shown in Figure S2A and B.

The network with these properties can be constructed using the method of Watts et al.[2]. Since our model consists of two-dimensional lattices with *N*=300 (15×20),

k=6 and *p*=0.2 are selected to attain the desired *L* and *C* under *N*=300. After constructing the SWN, nodes with over eight edges are regarded as hub nodes, which are located at random in the central region of the SCN lattice (Fig. S2C). Other nodes are then randomly scattered everywhere remaining, conserving all of the SWN connections. All edges of the SWN are described in the third term of our model as follows;

$$\frac{d\varphi_i}{dt} = \omega_i + g_{NN}\sum_j^{NN}\sin(\varphi_j - \varphi_i) + g_{SWN}\sum_j^{sw}\sin(\varphi_j - \varphi_i) \qquad (4)$$

where the summation in the third term represents the connections of the SWN, and g_{SWN} is the coupling constant which differs from g_{NN}.

Supplementary References

1. Barabási, A. L. *Network Science* (Cambridge University Press, 2016).

2. Watts, D. J. & Strogatz, S. H. Collective dynamics of ‘small-world’ networks. *Nature* **393**, 440-442 (1998).

Supplementary Figure legends

Supplementary Figure S1

The behavioral circadian rhythm in *Clock*Δ19 mutant mice on an ICR strain. (A) Representative double-plotted actogram of *Clock*Δ19 homozygous (right) and wild-type (left) mice in the light-dark cycle (LD) and constant darkness (DD) conditions. The top bars indicate the LD schedule and the upper numerals are the time of day. The white and gray backgrounds indicate the light and dark periods, respectively. The numerals on the right side show the number of days in the DD. (B) The free-running period lengths of *Clock*Δ19 homozygous and wild-type mice, which were calculated based on a one week interval indicated by a vertical line in (A). (C) Daily distribution of spontaneous activity of *Clock*Δ19 homozygous and wild-type mice in the final 2 days under the LD before transfer to the DD. The vertical axis is the percentage of the daily total activity. The top bar indicates the LD schedule. Means ± SEM, Tukey-Kramer test, *$p<0.05$, **$p<0.01$, ***$p<0.001$.

Supplementary Figure S2

Numerical simulation of the phase pattern with the small-world network (SWN). (A) An example of the SWN constructed by Watts-Strogatz method, with an

average path length of L=4.0614 and a clustering coefficient of C=0.3202. (B) A histogram of the edges is shown. Although the mean edge number remains around 6, because k=6, hub nodes with many edges also appears. In our model, the hub node is defined as one with over 8 edges, which are randomly distributed in the central region. (C) A schematic representation showing the arrangement of grid elements representing limit cycle oscillators. Short period oscillators (SPOs) were located in the short period region (SPR), which was in the medial small area. In addition, hub nodes in the SWN are distributed in the central region, appearing as dark grey. (D and E) Alteration to the phase difference by changing the coupling constant of the SWN and the nearest neighbor (nn). All simulations were carried out under the same conditions, except for the coupling constant. (F, G, H and I) Effect of the SWN on the phase difference by changing the localization rate of the SPR (lr), coupling constant of nearest neighbor (cc), synchronized period (p), and variance of each oscillator period (sd), respectively. The white and black bars indicate the phase difference without and with the SWN, respectively. The coupling constant of the SWN was set to 0.001. Data is represented as the relative value to the standard condition without SWN (lr=100, cc=0.2, p=23.1, and sd=1.0). Means ± SEM, Steel-Dwass test, *p<0.05, **p<0.01, ***p<0.01.

Figure S1

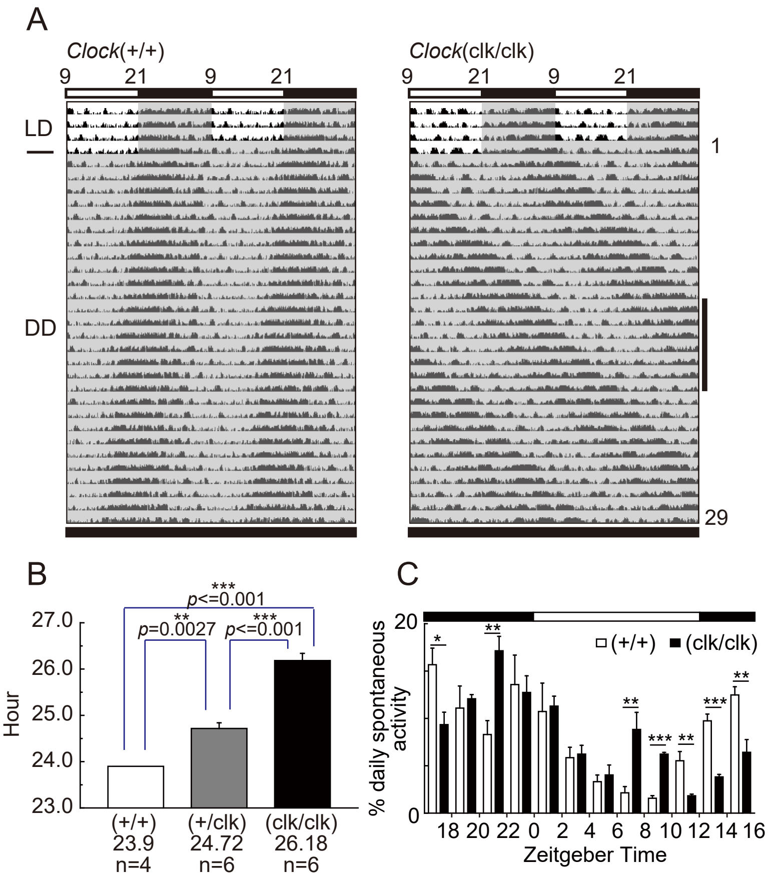
A
Clock(+/+)
Clock(clk/clk)
9
21
9
21
LD
DD
1
29
B
***
p<=0.001
**
p=0.0027
***
p<=0.001
27.0
26.0
25.0
24.0
23.0
Hour
(+/+)
23.9
n=4
(+/clk)
24.72
n=6
(clk/clk)
26.18
n=6
C
20
0
% daily spontaneous activity
(+/+)
(clk/clk)
18 20 22 0 2 4 6 8 10 12 14 16
Zeitgeber Time

Figure S2

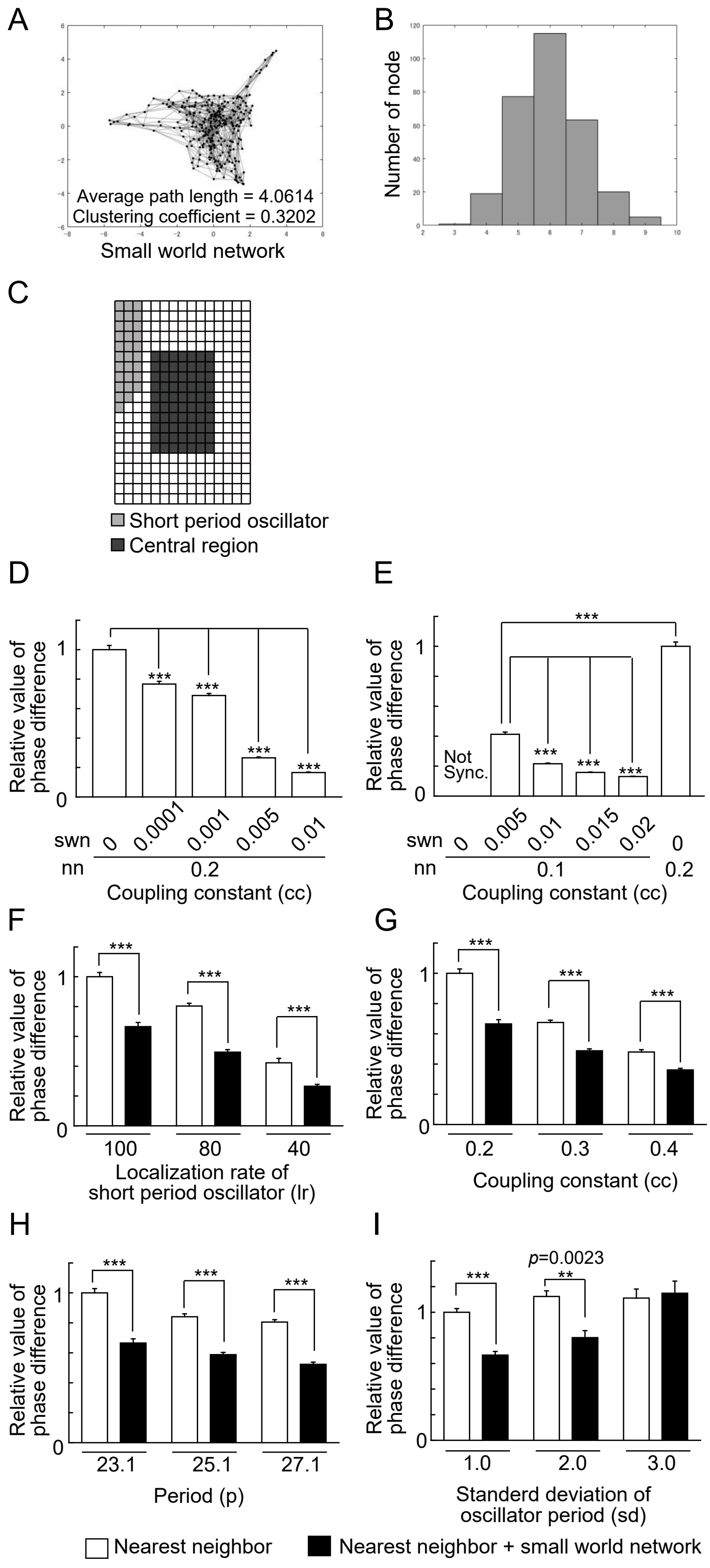
A
Average path length = 4.0614
Clustering coefficient = 0.3202
Small world network
B
Number of node
C
Short period oscillator
Central region
D
Relative value of phase difference
swn 0 0.0001 0.001 0.005 0.01
nn 0.2
Coupling constant (cc)
E
Not Sync.
swn 0 0.005 0.01 0.015 0.02 0
nn 0.1 0.2
Coupling constant (cc)
F
100 80 40
Localization rate of short period oscillator (lr)
G
0.2 0.3 0.4
Coupling constant (cc)
H
23.1 25.1 27.1
Period (p)
I
p=0.0023
1.0 2.0 3.0
Standerd deviation of oscillator period (sd)
Nearest neighbor
Nearest neighbor + small world network
